# *Lactobacillus paracasei* CBA L74 Metabolic Products and Fermented Milk for Infant Formula Have Anti-Inflammatory Activity on Dendritic Cells *In Vitro* and Protective Effects against Colitis and an Enteric Pathogen *In Vivo*

**DOI:** 10.1371/journal.pone.0087615

**Published:** 2014-02-10

**Authors:** Elena Zagato, Erika Mileti, Lucia Massimiliano, Francesca Fasano, Andrea Budelli, Giuseppe Penna, Maria Rescigno

**Affiliations:** 1 Department of Experimental Oncology, European Institute of Oncology, Milan, Italy; 2 R&D, Heinz Italia S.p.A., Latina, Italy; CWRU/UH Digestive Health Institute, United States of America

## Abstract

The rapid expansion of commercially available fermented food products raises important safety issues particularly when infant food is concerned. In many cases, the activity of the microorganisms used for fermentation as well as what will be the immunological outcome of fermented food intake is not known. In this manuscript we used complex *in vitro*, *ex-vivo* and *in vivo* systems to study the immunomodulatory properties of probiotic-fermented products (culture supernatant and fermented milk without live bacteria to be used in infant formula).

We found *in vitro* and *ex-vivo* that fermented products of *Lactobacillus paracasei* CBA L74 act via the inhibition of proinflammatory cytokine release leaving anti-inflammatory cytokines either unaffected or even increased in response to *Salmonella* typhimurium. These activities are not dependent on the inactivated bacteria but to metabolic products released during the fermentation process. We also show that our *in vitro* systems are predictive of an *in vivo* efficacy by the fermented products. Indeed CBA L74 fermented products (both culture medium and fermented milk) could protect against colitis and against an enteric pathogen infection (*Salmonella* typhimurium). Hence we found that fermented products can act via the inhibition of immune cell inflammation and can protect the host from pathobionts and enteric pathogens. These results open new perspectives in infant nutrition and suggest that *L. paracasei* CBA L74 fermented formula can provide immune benefits to formula-fed infants, without carrying live bacteria that may be potentially dangerous to an immature infant immune system.

## Introduction

In the initial years of human life the microbiota is established and several environmental factors contribute to its generation, including nutrition [1], [2]. The microbiota then plays a major role in the development of the immune system [3], [4]. Thus the interplay between nutrition, microbiota and immune cells is decisive for the subsequent health of the infant [5].

Exclusive breast-feeding is recommended from birth to 6 months of age [6], [7]. Breast milk is involved in the development of a bifidobacteria-enriched microbiota [8], in protection against potentially infectious agents [9] and the correct development of secondary lymphoid organs and the immune system [10]. Infant formulae are intended to serve as a substitute for breast milk in infants who cannot be fed at breast, should not receive breast milk, or for whom breast milk is not available [11].

It is known that infant formula does not recapitulate many of the effects of breast-feeding as it does not impact on thymus size [10] or microbiota composition [8].

Although human milk represents the gold standard, at present no infant formula is able to reproduce the whole composition and daily variability of human milk. Recent developments in infant formulae have therefore been targeted towards reproducing the functional effects, rather than the qualitative composition, of human milk [12].

Even though more evidence on clinical trials is required, fermented infant formula (FIF) without live bacteria has been shown to be beneficial for several aspects of the well-being of infants [13]. It has been shown that FIF administration in infants can increase the amount of bifidobacteria in the feces and fosters the development of poliovirus-specific IgAs in response to Pentacoq vaccination, as compared to infants treated with standard non-fermented formula (SF) [14]. Another study compared three groups of term neonates: 30 newborns were breast-fed and 60 were randomized to receive either FIF or SF. Infants were followed up for 4 months. They showed that breast-fed and FIF treated infants had comparable thymus indexes and lower fecal pH than SF-treated infants, suggesting that FIF may have similar effect to maternal milk [15]. Another study tested the effect of long-term administration on 971 infants (4–6 months of age) of FIF or SF with a similar nutritional composition on incidence or severity of acute diarrhea [16]. They found that there was no difference in the number or duration of diarrhea episodes, but there was a reduction in the severity of the episodes in the FIF group, including fewer medical consultations and fewer changes to other formulas [16]. Similarly, a randomized clinical trial compared long term treatment with FIF or SF of newborns with high risk of allergy until the age of 1 year old. While there was no difference in the proportion of infants affected by cow's milk allergy, infants administered FIF had reduced positive skin prick tests to cow's milk. FIF seems to be beneficial also in pre-term infants. While there was no major difference in the anthropometric data, pre-term infants treated with FIF displayed reduced abdominal distension from the second week of treatment [17]. Differently from term infants, there was no bifidogenic property of the FIF and secreted IgAs were increased in FIF treated infants, but only if the infants had been partially breast-fed, suggesting that FIF can boost the maternal IgA response [17].

Hence, FIF has been shown to be safe, to have similar nutritional properties of standard formula, and to mimic some effects of maternal milk, *in vivo*. However, several bacteria can be used to ferment milk, including probiotics, bacteria considered to be beneficial to the host. However, the choice of probiotics to be used should not be arbitrary as each strain may produce specific metabolites in the fermented product with distinctive immunomodulatory properties [18]. For instance, when we compared the activity of several *Lactobacilli* and their metabolic products - that we called postbiotic [19] - on the immune system, we found that each strain has very peculiar characteristics [19], [20], [21]. We found that *Lactobacillus plantarum* v299 has detrimental effects due to its immunostimulatory properties in mice and on healthy human intestinal tissue [20], [21], while LGG and *L. paracasei* B21090 can be detrimental on inflamed tissues like those coming from patients with inflammatory bowel disease [20]. By contrast, the activity of postbiotics is very safe also on inflamed tissues presumably because postbiotics lack the microbe associated molecular patterns that may further activate inflamed tissues [20].

In this manuscript, in the attempt to define the immunomodulatory properties of fermented milk powder preparations for infant formula without living bacteria, we analyzed the activity of metabolic products released by *Lactobacillus paracasei* CBA L74 during fermentation of culture media or milk for infant formula. We studied their properties *in vitro*, on one of the most important immune target (the dendritic cells, DCs) that are the initiators of immunity or tolerance; *ex-vivo*, using an organ culture model set up in our laboratory and *in vivo* in mice, during inflammation (colitis) or infection (*Salmonella* infection).

## Materials and Methods

### Mice and bacterial strains

C57/BL6 mice were purchased from Charles River laboratories. All mice were maintained in microisolator cages in a specific pathogen-free animal facility. All experiments were performed in accordance with the guidelines established in the Principles of Laboratory Animal Care (directive 86/609/EEC) and approved by the Italian Ministry of Health.

*Lactobacillus paracasei* CBA L74 (Heinz Italia SpA, Latina, Italy), International Depository Accession Number LMG P-24778 was grown in MRS broth (Biokar diagostics) in semi-anaerobic conditions.

*Salmonella enterica* serovar typhimurium strain SL1344 (FB62) was provided by G. Dougan (The Wellcome Trust Sanger Institute, UK) and grown in LB broth.

CFU numbers were controlled by plating.

*Lactobacillus paracasei* CBA L74 and *Salmonella* FB62 supernatants were obtained growing bacteria to OD_600_ = 0,6 in MRS and LB respectively and filtering the resulting medium.

### Cells and reagents

DCs were derived from human peripheral blood monocytes selected with anti-CD14 antibodies coupled to magnetic beads (Miltenyi, Bologna, Italy). CD14+ cells were incubated for 6 days in complete medium containing granulocyte-macrophage colony stimulating factor (GM-CSF, 5 ng/mL; BD Biosciences) and interleukin-4 (2,5 ng/mL; BD Biosciences) in order to obtain immature MoDCs.

MoDCs were incubated with either *Salmonella* FB62 (MOI 10∶1 bacteria:DC) or *Lactobacillus paracasei* CBA L74 (MOI 10∶1) for 1 hour in the presence of 2% LB, MRS, CBA L74 supernatant (in MRS) or FB62 supernatant. After extensive wash cells were incubated for 24 hours in the presence of gentamicin (100 µg/ml). Supernatants were tested for cytokine abundance and cells were analyzed by FACS for activation and maturation markers: HLA-DR FITC (BD) and CD83 APC (BD) respectively.

### Milk powders

Milk products in powder were provided by HJ Heinz (Heinz Italia SpA, Latina, Italy).

The fermented milk was prepared from skim milk fermented by *Lactobacillus paracasei* CBA L74 (Heinz Italia SpA, Latina, Italy), International Depository Accession Number LMG P-24778. The fermentation is started in the presence of 10^6^ bacteria, reaching 10^8^ colony-forming units/ml after a 15-hour incubation at 37°C. After heating at 85°C for 20 seconds, in view of inactivating the live bacteria, the formula is spray-dried. The final fermented milk powder (FM) contains bacterial bodies and fermentation products.

The control (non-fermented milk, NFM) consists of skimmed milk powder with the same basal nutrient composition of fermented milk powder (grams per 100 g): proteins, 35; lipids, 1; carbohydrates, 54.

Milk powders were resuspended at 1,41 g/100 ml in boiling water.

### Preparation of the control and fermented milk diets

The diets (control and fermented milk) had similar nutrient composition and were prepared as pellets at Mucedola, Settimo Milanese, Italy. The diets were a mixture of 95% standard rat/mice chow ref. 4RF21 (Mucedola, Settimo Milanese, Italy) and 5% of the fermented milk or non-fermented milk, respectively.

### *Ex-vivo* organ culture

The protocol developed in the lab by Tsilingiri K. et al. [20] for human intestinal explant culture was modified for murine colon tissue. Briefly: 25 mm^2^ chunks of murine colon were fixed to the cylinder. Tissue was incubated with non-fermented and fermented milk inside the cylinder for 1 hour at 37°C 5% CO_2_ and then infected with 10^2^ CFU FB62 resuspended in 50 µl of the respective preparation. After 2 hours at 37°C 5% CO_2_ infection medium was removed and gentamicin (100 µg/ml) added in the culture medium. Tissues were incubated for 24 hours in oxygen chamber, then culture media were analysed for cytokine production and tissues fixed for paraffin embedding and histological evaluation.

### DSS colitis

Mice were administered intragastrically for 14 consecutive days (from day -2 to day 11) with 200 µl of vehicle, MRS, live CBA L74, supernatant of CBA L74, non-fermented milk or fermented milk. When special diets were used mice were fed with modified diet from day −10 until the end of the experiment.

Acute colitis was induced supplementing drinking water with DSS 3% (w/v) for 8–9 days. Recovery in plain water was allowed until the end of the experiment.

Body weight and survival were assessed daily.

### *Salmonella* survival

After receiving modified diet for 10 days mice were intragastrically infected with 10^6^ CFU FB62 in 200 µl carbonate buffer. Survival was assessed daily.

### Histological analysis

4 µm sections from colon swiss rolls were stained with Hematoxylin-Eosin (HE) standard protocol. Histological evaluation of the grade of colitis was performed according to the following histological grading system: 0 normal colonic mucosa; 1 minimal to mild inflammation restricted to the mucosa; 2 moderate inflammation in the mucosa, only occasionally extending to the submucosa; 3 moderate to severe inflammation involving the mucosa with shortening or loss of the crypts, extending into the submucosa and occasionally into the *tunica muscularis*; 4 severe transmural inflammation in the mucosa, submucosa, *tunica muscularis*, and subserosa, usually associated with mucosal ulceration. Additionally, the number of lymphoid aggregates was counted and the grade of colon edema (0 = absent; 1 = minimal; 2 = mild; 3 = moderate; 4 = severe) was evaluated. For each colon also the total number of ulcers and the number of healing ulcers (i.e. re-epithelialized ulcers) were counted. According to their size, ulcers were classified as follows: small  =  <10 crypts (or <1 field at 400x); medium  = 10–20 crypts (<1 field at 100x) in width; large  = >20 crypts (>1 field at 100x) in width. Arbitrarily, a value of 1 was assigned to each small ulcer, a value of 2 to each medium ulcer, and a value of 4 to each large ulcer. The total ulcer score was then calculated for each colon as follows: Total ulcer score  =  no. of small ulcers*1+ no. of medium ulcers*2+ no. of large ulcers*4.

### Cytokine measurements

IL-10, IL-6, KC and TNF-α concentrations were determined by Cytokine bead array (Becton Dickinson) and IL-12p70 concentration was determined by commercially available ELISA (R&D systems). Optical densities were measured on a Bio-Rad Dynatech Laboratories ELISA reader at a wavelength of 450 nm (Hercules, CA, USA). CBA-associated Cytofluorimetry was measured by FACS array (Becton Dickinson).

Anti-inflammatory index was calculated as ratio between IL-10 and IL-12% of response relative to FB62 stimulation.

### RT-qPCR assay

Colons were homogenised in 500 ul of TRIzol (Invitrogen). RNA was extracted adding 100 ul of chloroform, precipitating the acqueous phase with 300 ul of 70% ethanol and purifying RNA with RNeasy Mini Kit (QIAGEN). RNA was retro-transcribed with ImProm-II Reverse Transcriptase kit (Promega) following manufacturer's instruction. qPCR assay was performed with Fast Sybr Green Master Mix (Life Technologies) on 10 ng cDNA template/reaction using exon-spanning primers. QuantiTect Primer Assays were used for Il22, Reg3γ and Reg3β. Other primers are listed in Table S1. The Spearman's rank correlation coefficient among cytokine expression levels and inflammatory parameters was determined using GraphPad Prism.

## Results

### Metabolic products from CBA L74 have a high anti-inflammatory index

**Figure 1:**
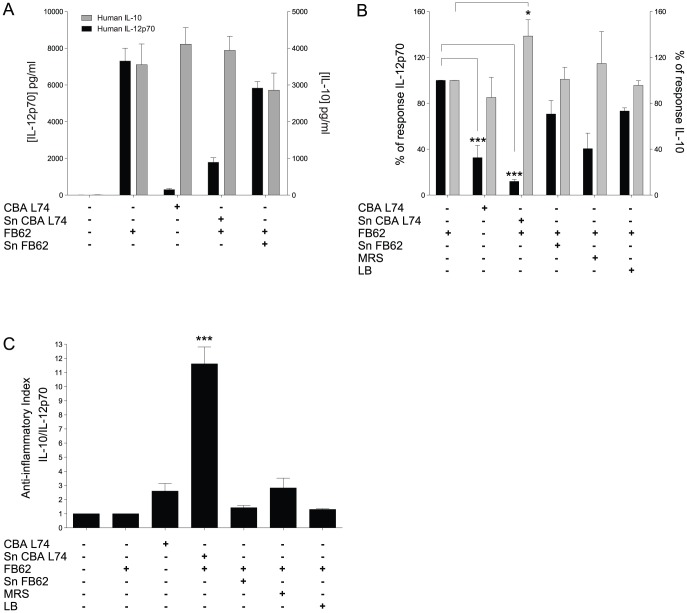
*Lactobacillus paracaseii* CBA L74 supernatant has anti-inflammatory properties on MoDCs. MoDCs were infected with either *Salmonella* SL1344 (MOI 10∶1) or *Lactobacillus* CBA L74 (MOI 10∶1) for 1 hour in medium without antibiotics or in the presence of 2% LB, MRS, FB62 supernatant (Sn FB62) or CBA L74 supernatant in MRS (Sn CBA L74). After washing and 24 h incubations in medium with antibiotics cytokine abundance was evaluated. A. Concentrations of IL-12p70 and IL-10 as determined by ELISA and CBA assays respectively. B. The % of response relative to FB62 infected MoDCs is shown after grouping six experiments together. C. Anti-inflammatory index calculated as ratio of IL-10/IL12p70 concentrations. * p<0,05; *** p<0,001.

Probiotics or their metabolic products can induce a particular type of cytokine profile depending on their inflammatory or anti-inflammatory properties [21], [22], [23]. However, when probiotics or their metabolic products have anti-inflammatory properties, their activity is best appreciated when they are tested concomitantly with a strong inflammatory agent [21]. To test the activity of *Lactobacillus paracasei* CBA L74, we incubated human monocyte derived dendritic cells (MoDCs) with either the live bacteria, or its supernatant of culture after centrifugation and ultrafiltration in the presence or absence of *Salmonella* typhimurium (FB62), which induces a strong inflammatory response in DCs [21]. As shown in Fig. 1A, while *Salmonella* induced a high production of the inflammatory cytokine IL-12p70 and of the anti-inflammatory cytokine IL-10, CBA L74 alone induced very little IL-12p70. Culture supernatant of FB62 alone, but not of CBA L74, induced some cytokine production by the MoDCs with higher IL-10 than IL-12p70 (Fig. S1). However when tested together with *Salmonella*, only the supernatant from CBA L74 had the ability to drastically reduce IL-12p70 and increase IL-10 (Fig. 1A-B). The net result was a strong anti-inflammatory index associated only to supernatant of CBA L74 when coincubated with *Salmonella* (Fig. 1C). By contrast, the same anti-inflammatory activity was not observed coincubating the DCs with *Salmonella* in the presence of the whole CBA L74 bacteria (Fig. S1B). The anti-inflammatory effect of CBA L74 sn was not due to inhibition of DC maturation (Fig. S1C).

Consistent with our previous results on a different *Lactobacillus* strain (B21090) [21], the activity was so strong that it was evident at a concentration of 2% of supernatant of bacterial culture, which is equivalent to the number of bacteria used to stimulate the DCs. This suggests that in the short period of stimulation before antibiotic killing, the bacteria are not able to release the metabolic products in a sufficient amount to observe the anti-inflammatory effect. Hence, metabolic products released by CBA L74 have strong anti-inflammatory activity on DCs stimulated with *Salmonella*.

### Metabolic products of CBA L74 protect against colitis *in vivo*

**Figure 2:**
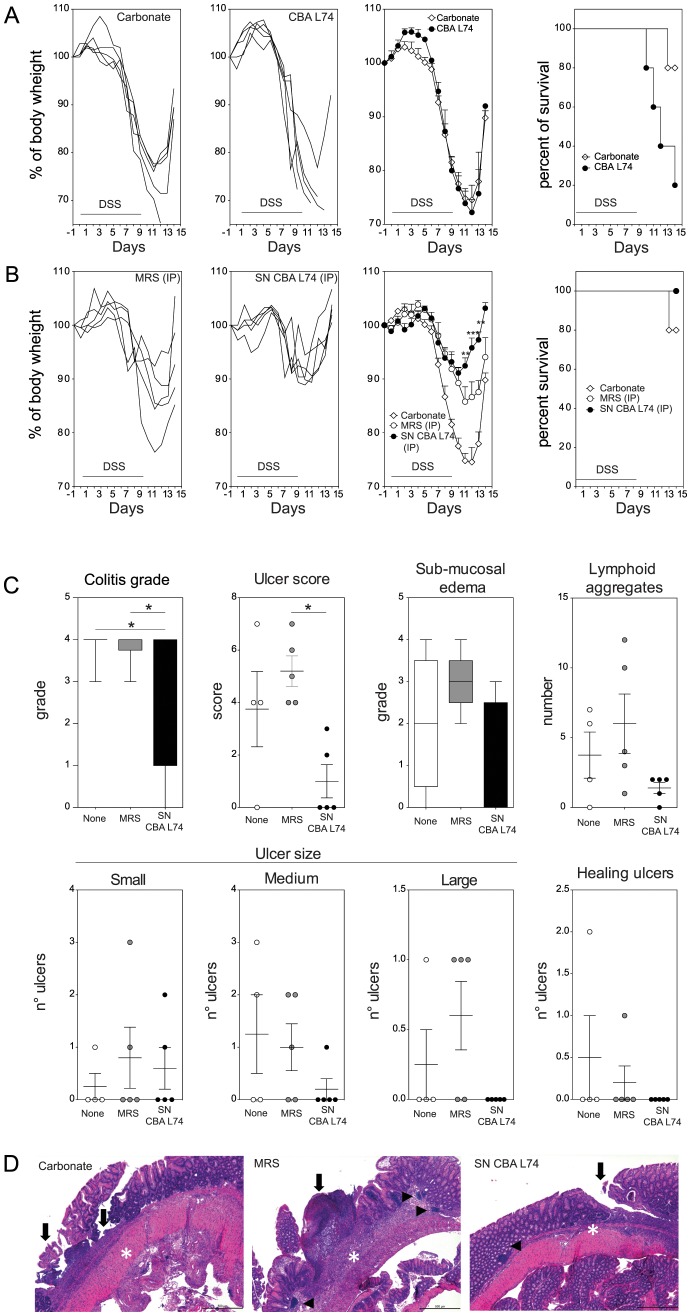
*Lactobacillus paracaseii* CBA L74 supernatant ameliorates DSS colitis when administered intraperitoneally. Mice (n = 5) were administered on a daily basis for 14 consecutive days (days −2 to 11) with carbonate or live CBA L74 (10^9^ CFU) intragastrically (ig) or with MRS or CBA L74 supernatant (SN CBA L74) intraperitoneally (ip). Acute colitis was induced by dissolving 3% DSS in drinking water for 9 days (days 0–9). Panels from left to right show weight curves of single mice, mean weight loss ad Kaplan-Meier survival for A. carbonate and live CBA L74 ig treated groups B. MRS and CBA L74 supernatant (SN CBA L74) ip treated groups. C. Histological parameters for carbonate, MRS ip and SN ip treated groups. D. Representative histologies for carbonate, MRS ip and SN ip treated groups. Arrows indicate ulcers, arrowheads lymphoid follicles and asterisks inflammation. Bar size  = 500 µm, magnification 50x. * p<0,05; ** p<0,01; *** p<0,001.

Probiotics have been proposed for the treatment or prevention of inflammatory bowel disease, however only in pouchitis and ulcerative colitis some beneficial effects were observed [18], [24]. We have shown that probiotics can be detrimental if administered to inflamed tissues *ex-vivo* probably because of boosting an ongoing inflammation, while postbiotics may be a safer alternative as they can inhibit current inflammatory responses [20]. Hence, we wanted to assess whether we could administer probiotics or postbiotics to mice in order to prevent colitis development. Mice were pretreated with either CBA L74 orally, or its culture supernatant or unfermented culture medium intraperitoneally for two days, and then received DSS into the drinking water to induce colitis while on treatment. We decided to use the intraperitoneal administration of the supernatant to avoid possible degradation of the metabolic products due to transit in the stomach acidic environment. Mice were followed for signs of colitis development (body weight and presence of blood in feces). At the end of the experiment mice were sacrificed and intestinal tissues were collected for histological analysis. As shown in Fig. 2A, administration of live bacteria to mice prior to DSS administration was highly detrimental, as mice lost weight similarly to untreated animals, but they all died in a few days. By contrast, intraperitoneal injection of CBA L74 fermented culture supernatant resulted in protection against colitis by reducing weight loss and favoring recovery of the mice (Fig. 2B). Also the unfermented culture medium had some protective effect, but the effect of the fermented medium was higher both as reduced weight loss, colitis grade, lymphoid aggregates and number and size of ulcers (Fig. 2C). Colitis score was assessed as described in material and methods section. The histological analysis indicated that the tissue of mice treated with CBA L74 fermented culture supernatant displayed a healthy morphology (Fig. 2D), whether this is due to a better recovery or to protection against the inflammatory effects of the DSS-induced colitis is difficult to ascertain at this time point. However, the finding that mice lost weight similarly to the MRS unfermented control medium but recovered faster would indicate that an effect on the recovery phase may have occurred.

Hence, we found that CBA L74 fermented medium has anti-inflammatory activity *in vitro* on DCs and has protective effect *in vivo* against colitis.

### Fermented milk powder preparations for infant formula have anti-inflammatory properties *in vitro*

**Figure 3:**
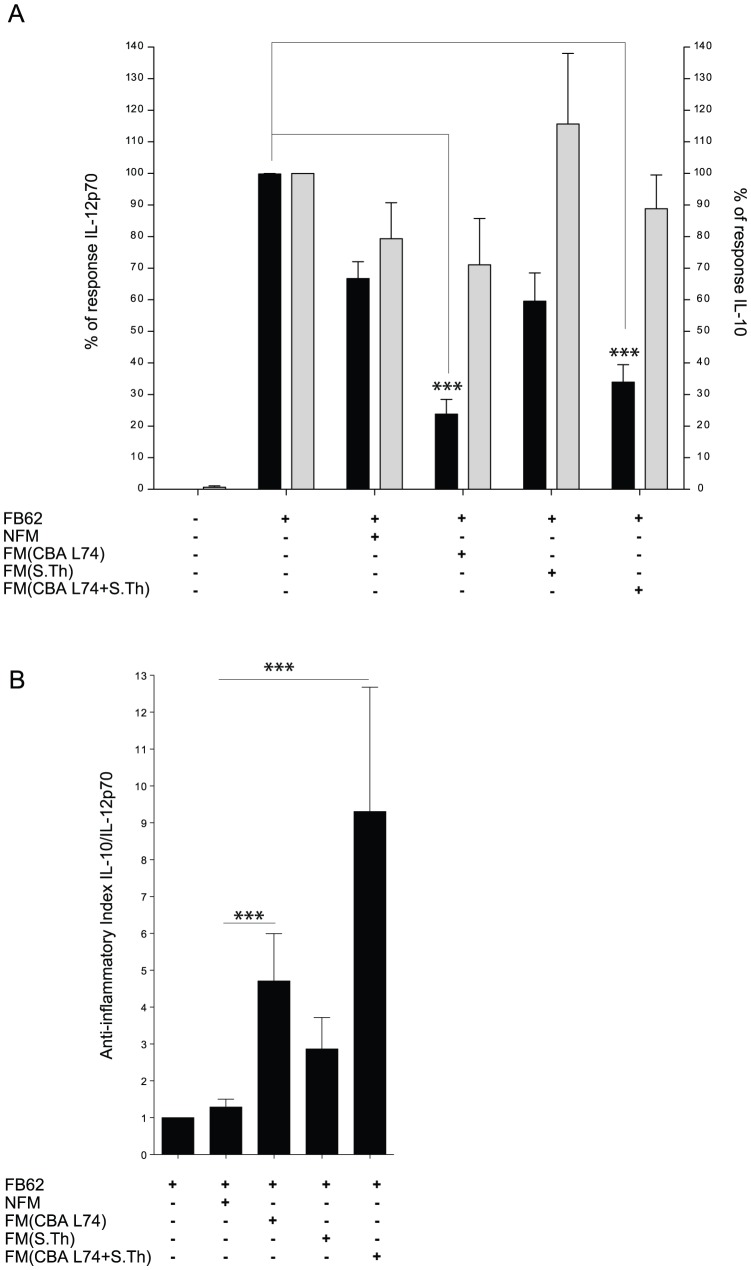
Fermented milk preparation has anti-inflammatory properties on MoDCs. MoDCs were infected with *Salmonella* FB62 (MOI 10:1) for 1 hour in medium without antibiotics or in different infant formulas: non-fermented milk (NFM) and fermented milk (FM) obtained through fermentation of Lactobacillus CBA L74 alone (CBA L74), fermentation in the presence *S. thermophilus* TH3 (S. th) or double fermentation with CBA L74 and *S. thermophilus* TH3 (CBA L74+S. th). After washing and 24 h incubations in medium with antibiotics cytokine abundance was evaluated. Panels show % of response of IL-12p70 and IL-10 relative to MoDCs infected with SL1334 in medium without antibiotics. *** p<0,001.

Having shown that CBA L74 fermentation leads to the generation of anti-inflammatory metabolic products that protect against colitis *in vivo*, we wanted to assess whether fermented food that may be administered to healthy infants also retains similar anti-inflammatory activity and whether this may result in protection against colitis or infectious diseases. We first tested whether reconstituted fermented milk powder preparations for infant formula (FM) had similar anti-inflammatory activity on the major immune cell targets that are the dendritic cells. To our knowledge this is the first attempt to assess which cell types may be the target of FM. MoDCs were incubated with reconstituted FM or non-fermented milk powder for infant formula (NFM) and/or a strong inflammatory infectious agent (*Salmonella* typhimurium) for 1 h and then thoroughly washed to eliminate any remaining milk and to reduce the bacterial burden. Cells were then incubated in a medium containing gentamycin to kill all the remaining *Salmonella*. We used two different fermented formulations, one containing only CBA L74 and another one with CBA L74 and *S. thermophilus*. While both formulations of FM alone had no major cytokine-inducing property (not shown), when coincubated with *S.* typhimurium, they both reduced IL-12p70 production while preserving the production of IL-10 by the dendritic cells (Fig. 3). The anti-inflammatory index was very high and significantly different from the one of the non-fermented milk (that was not different from *Salmonella* alone) for both FM formulations (Fig. 3). The two FM formulations did not statistically differ, even though the one obtained with CBA L74 and *S. thermophilus* had greater anti-inflammatory index. The anti-inflammatory activity of the FM was not due to the dead bacteria present in the preparation as NFM added with heat killed bacteria had no anti-inflammatory properties (not shown).

### Fermented milk for infant formula has protective effect against colitis *in vivo* when administered orally

**Figure 4:**
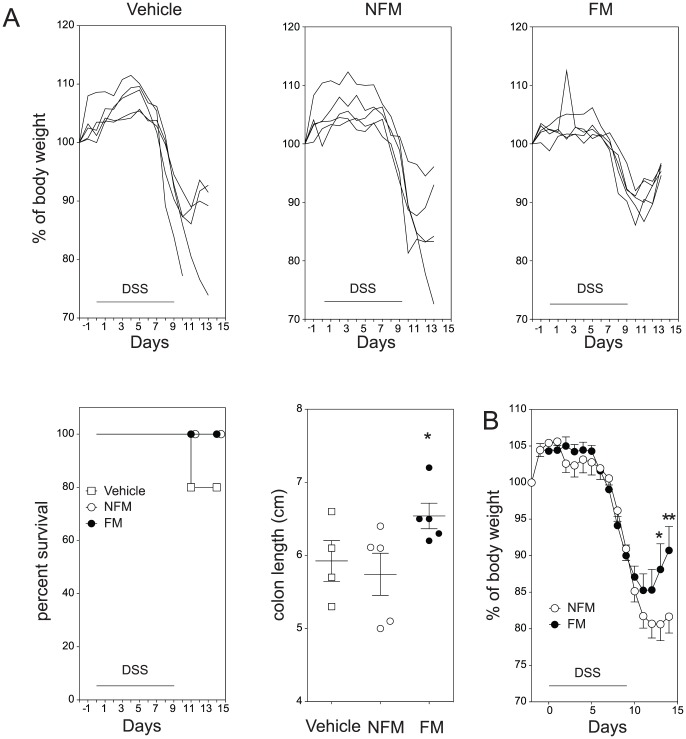
Oral administration of fermented milk preparation protects against colitis. Mice (n = 5) were orally administered on a daily basis for 14 consecutive days (days −2 to 11) with vehicle (water), non-fermented milk (NFM) or fermented milk (FM). Acute colitis was induced by dissolving 3% DSS in drinking water for 9 days (days 0–9). A, Single mouse body weight curves, Kaplan-Meier survival and colon length for vehicle, NFM and FM. B. Body weight curves (mean ± SEM) for two pooled experiments. * p<0,05; ** p<0,01.

**Figure 5:**
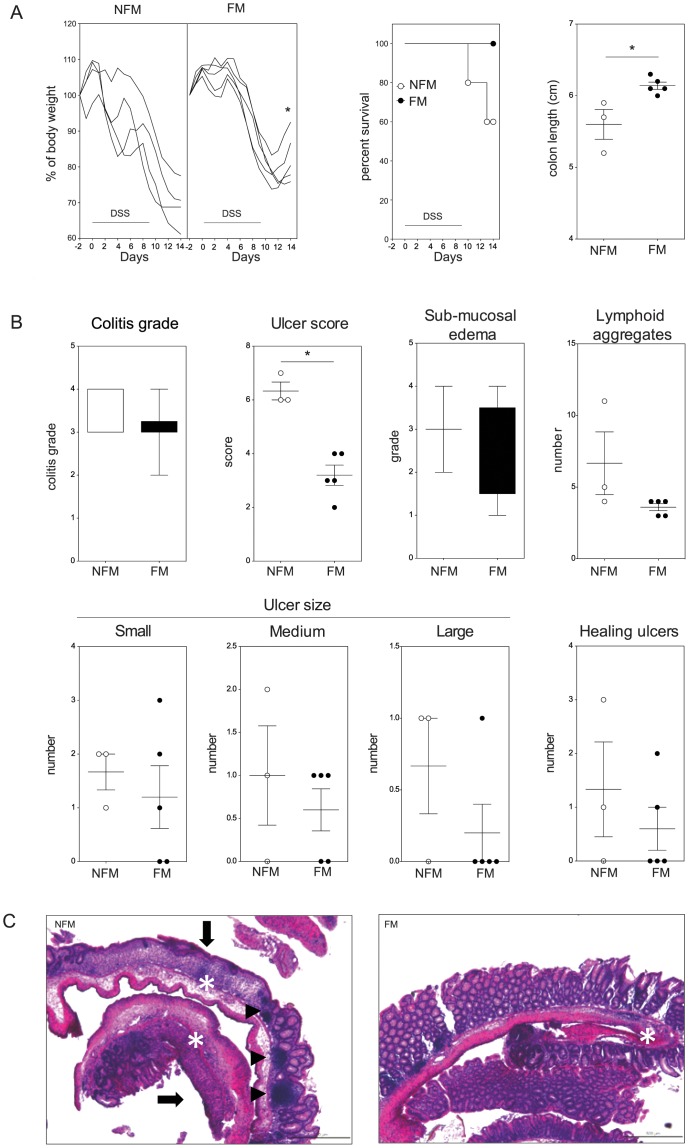
Oral administration of CBA L74 fermented milk preparation protects against colitis. Mice (n = 5) were orally administered on a daily basis for 14 consecutive days (days −2 to 11) with non-fermented milk (NFM) or milk fermented with only CBA L74 (FM). Acute colitis was induced by dissolving 3% DSS in drinking water for 9 days (days 0–9). A. Single mouse body weight curves, Kaplan-Meier survival curve and colon length for NFM and FM. B. Histological parameters. C. Representative histologies for NFM and FM. Arrows indicate ulcers, arrowheads lymphoid follicles and asterisks inflammation. Bar size  = 500 µm, magnification 50x. * p<0,05.

As shown earlier, DCs can be the target of the anti-inflammatory activity of FM *in vitro*, similarly to what observed with fermented culture medium. We then wondered whether also FM had colitis protective effects *in vivo*. Mice received FM (fermented with CBA L74 and *S. thermophilus*) or NFM by oral gavage for 14 consecutive days (from day −2). Starting from day 0 they also received DSS in their drinking water for 9 days. Mice were analyzed for development of colitis as reported above. As shown in Fig. 4A–B, mice receiving FM lost less weight and recovered faster than mice receiving vehicle or NFM. The mice all survived DSS but the colon length was much higher for mice receiving FM. Similar results were obtained with a preparation of FM obtained only with CBA L74 fermentation (in the absence of the starter *S. thermophilus*) indicating that the protective effects were due to the metabolic activity of CBA L74 (Fig. 5A). In addition, while the colitis score was very similar between the two groups, mice receiving FM had much reduced numbers and size of ulcers and lymphoid aggregates (Fig. 5B) and showed a better recovery of the morphology of the tissue, as shown by histological analysis (Fig. 5C).

**Figure 6:**
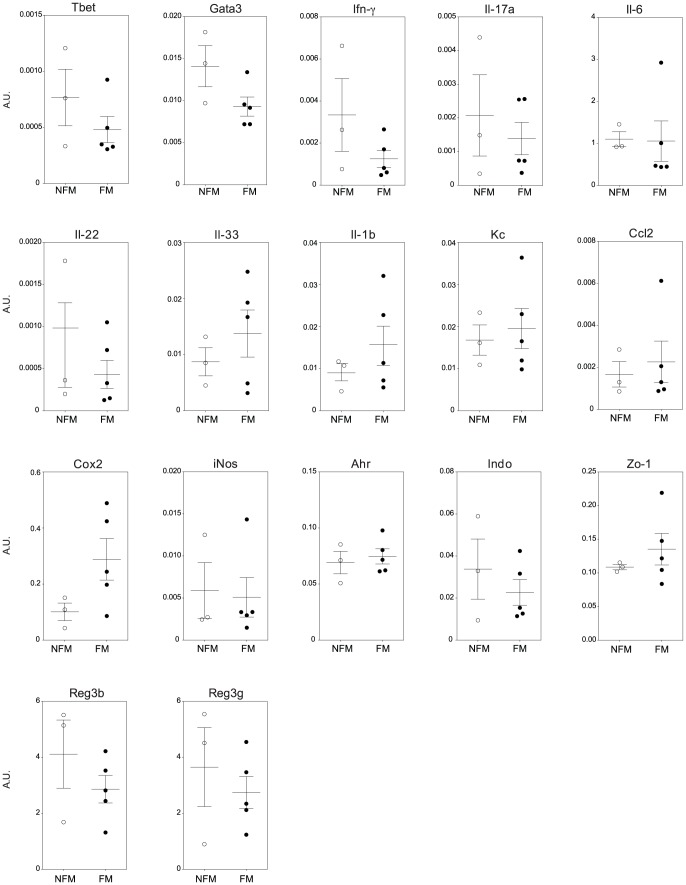
CBA L74 fermented milk reduces inflammatory mediators expression. At the end of the experiment described in Figure 5 RNA was extracted from colons and expression levels of Tbet, Gata3, Inf-γ, Il-17a, Il-6, Il-22, Il-33, Il-1β, Kc, Ccl2, Cox2, iNos, Ahr, Indo, Zo-1, Reg3β, Reg3γ were assessed by RT-qPCR. Expression levels are normalized to the housekeeping mRNA Ralp32. Dots represent individual mice measurements, lines represent the average value.

We then analyzed whether we could observe differences in cytokine expression in the intestines of mice pretreated with FM or NFM that may explain the protective effect of FM. We found no statistically significant differences in the two groups, but there was a tendency to a reduction in Th1 polarization (as attested by reduced expression of Tbet and IFN-γ in intestinal cells) (Fig. 6). There was also a slight reduction in IL-17a and Gata-3 expressing cells. Interestingly, we observed a tendency towards an increase in IL-33 and Cycloxygenase (Cox)-2 production in the FM group, indicating that there is a concomitant reduction of pathologic cytokines (IFN-γ and IL-17a) and increase of protective factors (IL-33 and Cox-2). However, when we analyzed the cytokine expression in relation to the inflammatory parameters, we found that some cytokines/transcription factors that were differentially expressed between the two groups but that did not reach statistical significance, were associated to the number of ulcers in a statistically significant way. In particular, IL-33 was inversely correlated to the number of ulcers (r Spearman −0.638, p = 0.048) while T-bet, Gata-3 and IFN-γ were directly correlated with the number of ulcers (r Spearman 0.792, p = 0.011).

As, for experimental difficulties, the amount of FM that was administered by gavage did not resemble the amount of FM an infant would introduce with the formula, we decided to prepare a special diet containing 5% of fermented milk. Mice were fed the FM or NFM-containing diet for 14 days as before and were treated with DSS starting from day 9 for 5 consecutive days. As shown in Fig. S2, also increasing the amount of fermented milk ingested via the diet we observed a drastic protection of mice from DSS colitis. While 90% of the mice died when exposed to NFM containing diet, only 30% of the mice died when fed with FM-diet and they all recovered (Fig. S2).

### FM for infant formula protects against a lethal dose of *Salmonella* typhimurium infection

**Figure 7:**
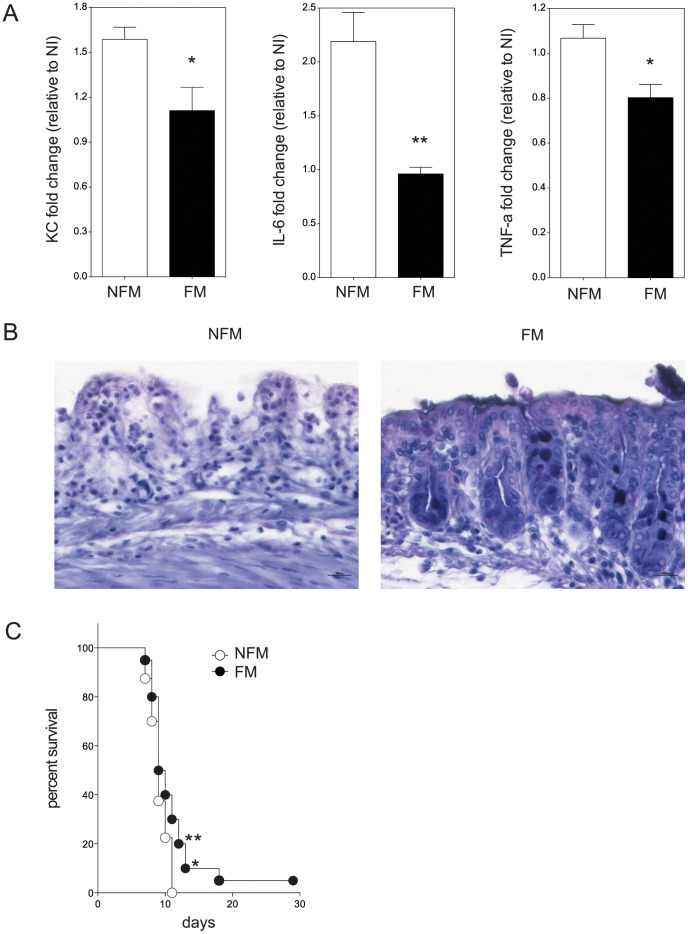
Fermented milk is protective against *Salmonella* infection in *ex vivo* and *in vivo* infection models. Murine colonic tissue was incubated with NFM of FM for 1 h at 37°C. Then tissue was either infected or not with *Salmonella* FB62 (10^2^ CFUs) for 2 hours. Tissue was then incubated with gentamicin for 24 h in oxygen chamber. A. Cytokine concentration culture media expressed relative to non-infected samples (NI). B. Representative histologies for NFM and FM treated samples infected with FB62, magnification 40×. C. Mice (n = 20) were fed with modified diet containing 5% of non-fermented milk (NFM) or fermented milk (FM). After 10 days mice were orally administered with 10^6^ CFUs of *Salmonella* FB62. Survival was monitored daily. Graph represents Kaplan-Meier Survival Probability Estimate and statistical significance was calculated with two-way ANOVA. * p<0,05; ** p<0,01.

An important characteristic a FM should have, when thinking to infant nutrition, is protection against enteric pathogens. One of the major pathogen of the mouse gastrointestinal tract is *Salmonella* typhimurium [25]. We wanted to test whether FM would protect against *S.* typhimurium infection. We adopted two strategies: an *ex-vivo* one, to strictly control the infection and to assess the direct effect of the FM on intestinal tissue in response to *Salmonella* and an i*n vivo* one, using a mouse model of infection. In the experiments *ex-vivo*, we used an organ culture model system established in our laboratory that we can use to stimulate in a polarized fashion [20]. In fact we glue a cave cylinder on the apical face of the intestinal mucosa that allows to confine the size of the infection and to avoid spreading of bacteria on the broken edges of the tissue, thus preserving tissue polarity and permitting physiological stimulation [20]. The cylinder is then excided and the tissue can be analyzed for histology and the supernatant for cytokine production. We found that FM could drastically reduce the amount of the inflammatory cytokines KC (the equivalent of human IL-8), IL-6 and TNF-α, produced in response to *Salmonella* and to limit tissue destruction (See Fig. 7A–B). In the experiments *in vivo*, we fed the mice with a FM-containing diet for ten days and then challenged the mice with a lethal dose of *S.* typhimurium. As shown in Fig. 7C, mice receiving FM-diet died with a statistically significantly slower kinetic than mice receiving NFM-diet, indicating a slight protection against a lethal dose of *Salmonella*.

## Discussion

In 2007 a concerted action between the world health organization (WHO) and the UNICEF defined a ‘Global Strategy for Infant and Young Child Feeding’ to raise awareness on the importance of a proper nutrition for infants till the age of 5 (http://www.who.int/nutrition/topics/global_strategy/en/). In this document it is highlighted the importance of facilitating mothers to exclusively breastfeeding in the first 6 months of life.

If, for whatever reason, human milk becomes unavailable to an infant, then during the first 6 months of life a new-generation cows' milk formula that is closer to the gold standard of human milk should be used. Infant formulae are the only alternatives to breast milk for infants who are unable to continue breastfeeding through the first year of life [12].

In order to mimic some benefits observed in breast-fed infants, functional ingredients such as fermented milk have been added to infant formulae in recent years.

Even though there are several reports on the use of fermented formula in infants, only a few studies have been carried out so far to understand which are the immune cell targets of fermented milk preparations (without live bacteria) for infant formula and what are their major effects *in vivo* in animals in order to understand their mechanisms of action. It has been shown that heat-treated fermented formula (*Bifidobacterium breve* C50 and *Streptococcus thermophilus* 065) does not interfere with the establishment of oral tolerance but it strengthens the epithelial barrier properties [26].

Here, we compared the immunological properties of fermented and non fermented milk for formula preparations. We found that bacterial culture medium or milk fermented with *L. paracasei* CBA L74 has strong anti-inflammatory properties on dendritic cells in response to the inflammatory enteric pathogen *Salmonella* typhimurium. This indicates that during fermentation, *L. paracasei* CBA L74 produces metabolites that play an important role in controlling the dendritic cell response to inflammatory agents. The DCs were capable of fully mature (both phenotypically and morphologically) but were producing less IL-12p70 in response to *Salmonella*, while IL-10 release was not affected. Overall, both CBA L74-fermented culture medium and milk displayed a potent anti-inflammatory index (the ratio between IL-10 and IL-12p70 production). We also tested if the dead bacteria (thus mimicking the effect of killing bacteria by heat after fermentation) may be responsible of the anti-inflammatory activity of the milk, but dead *L. paracasei* was not influencing the inflammatory activity of *Salmonella*. This has led us to test the effect of these metabolic products also *in vivo* in mice after colitis induction or infection.

Interestingly, we found that CBA L74- fermented culture medium could contrast the onset of colitis (there was a statistically significant reduction in weight loss) and favor the recovery of mice thus completely protecting them from colitis-induced death. Of note, while the administration of fermented milk was protective, the administration of live bacteria was detrimental, leading to mice death. This has important implications when considering the use of fermented formulas containing live probiotics, as these may be dangerous when administered to a non-immune infant host.

When we tested the properties of fermented milk, we found that it displayed protective effects against colitis when administered intragastrically and this correlated with a reduced expression, which was however not statistically significant, of the pathologic Th1 cytokine IFN-γ in the colons of treated mice. We also observed a tendency to IL-33 increased production in mice fed with FM. IL-33, a member of the IL-1 family, signals via the ST-2 receptor and is responsible for the polarization of Th2 T cells [27]. It is upregulated in patients with inflammatory bowel disease [28], however whether it has pathological or protective effects is still a matter of debate. Indeed, in some models of colitis the absence of the IL-33/ST-2 axis is protective, suggesting a pathologic role of IL-33 [29], [30]; while in others the absence of IL-33 results in reduced recovery and mouse death, indicating a protective role [31], [32]. Interestingly, the protective effect of IL-33 seems to be exerted in the recovery phase [32], presumably via the activation of Foxp3+ regulatory T cells [31]. Because we found an increase in IL-33 during the recovery phase in the FM-treated group, it is likely that this cytokine has a protective function in this phase, however as this difference was not statistically significant we cannot consider it as directly related to the overall anti-inflammatory effect exerted by the fermented milk. We also observed an increased but not statistically significant expression of Cox-2 in the FM-treated group. Global deletion or chemical inhibition of Cox-2 results in exacerbated colitis [33], [34], suggesting a protective role for Cox-2 during intestinal injury. Hence, small differences towards reduction of pro-inflammatory IFN-γ producing cells and increase of protective cytokines (IL-33) or enzymes (Cox-2) may be responsible for the overall protective effect of FM against colitis and may not reach statistical significance when considered individually. The colitis-protective effect was observed also when the fermented milk powder was added to the pellet diet. By contrast the non-fermented milk did not display any protective effect suggesting that the fermentation in itself is responsible for the observed effect.

Another important characteristic of breast milk is that it can protect against enteric pathogens. This characteristic is often associated to the IgAs that are passively delivered to the pups via the milk [35], [36]. However, it is also possible that milk-associated microbiota [37] or its metabolic products may reinforce the epithelial barrier or the immune system against pathogens. By comparing mice fed with fermented or non-fermented milk preparations, we could address this issue as there are no mother-derived IgAs, and analyze if fermented milk could protect against enteric infections. We found that mice fed with the fermented milk preparation displayed a slightly longer survival to a lethal dose of *Salmonella*. This indicates that *L. paracasei* CBA L74 metabolic products released in the milk have enteric pathogen protective effects. Indeed, the exposure of colon explants to a fermented milk preparation resulted in a reduced capacity of the pathogen to cause inflammation and tissue destruction. Whether the latter effect is a consequence of the reduced inflammation or the reduced capacity of *Salmonella* to infect the tissue remains to be established. We also do not know whether the fermented products are strengthening the barrier properties of the epithelium besides reducing the inflammation.

The capacity of postbiotics to protect against colitis or pathogen infection has been reported also for other bacterial strains. *Lactobacillus brevis* SBC8803 derived polyphosphate is capable of protecting mice against DSS-induced colitis, acting on the p38 MAPK pathway and suppressing intestinal permeability induced by oxidants [38]. The p40 molecule, produced and secreted by *Lactobacillus* GG, is the main mediator for ameliorating DSS and oxazolone-induced inflammation, through its binding to EGFR. Activation of EGFR by p40 was sufficient to reduce cytokine-induced IEC apoptosis *in vitro* and *ex-vivo* [39]. *Lactobacillus gasseri* OLL 2716 and *Propionibacterium freudenrichii* ET-3 fermentation metabolites can increase the number of CD4 and CD8 lymphocytes in blood and the capacity of macrophages to produce nitric oxide and reactive oxygen species [40]. Consistently, we have observed that *L. paracasei* strain B21060 has preventive effect in the DSS colitis model [21], but is detrimental when applied on intestinal mucosal explants from IBD patients, whereas the culture supernatant exerts a prominent anti-inflammatory effect on the explants from the same patients [20]. However, to our knowledge this is the first study addressing the effect of fermented milk metabolites on dendritic cells or on inflammatory responses *in vivo*. We have not yet identified, the compound/s of the culture supernatant or of the fermented milk having the protective effect and we also do not know whether they are the same in the two fermentations. Further studies are needed to characterize the nature of the compound/s and to identify their molecular structure.

In conclusion, we have shown that *L. paracasei* CBA L74 fermented milk preparations for infant formula have a strong anti-inflammatory activity *in vitro* and protect against colitis or enteric pathogens *in vivo*. This opens new perspectives in infant nutrition and suggests that *L. paracasei* CBA L74 fermented formula can provide some immune benefits to formula-fed infants, without carrying potentially dangerous live bacteria.

## Supporting Information

Figure S1 A. MoDCs were incubated for 1 h with CBA L74 supernatant (Sn CBA L74), FB62 supernatant (Sn FB62), MRS or LB. After washing and 24 h incubations, concentrations of IL-12p70 and IL-10 were determined by ELISA and CBA assays. Concentrations are shown as % of response relative to untreated MoDCs. B. MoDCs were infected with either *Salmonella* FB62 (MOI 10∶1) or *Lactobacillus* CBA L74 (MOI 10∶1) for 1 hour in medium without antibiotics or in the presence of CBA L74 supernatant (Sn CBA L74). After washing and 24 h incubations in medium with antibiotics, cytokine abundance was evaluated. C. MoDCs were infected for 1 h with either *Salmonella* FB62 (MOI 10∶1) or *Lactobacillus* CBA L74 (MOI 10∶1) for 1 hour in medium without antibiotics, washed, incubated for 24 h and analysed at FACS for CD83 and HLA-DR surface markers. * p<0,05; *** p<0,001.
(EPS)

Figure S2 Diet containing fermented milk protects against colitis. Mice (n = 10) were fed with modified diet containing 5% of non-fermented milk (NFM) or fermented milk (FM). After 10 days acute colitis was induced by 9-day administration of DSS 3% in drinking water. Panels show single mouse weight curves, weight curves (mean ± SEM) and Kaplan-Meier survival for NFM and FM. * p<0,05; *** p<0,001.
(EPS)

Table S1 List of forward and reverse primers used for Q-PCR of the reported genes.
(PDF)
