# Supplementary material for: Lactobacillus paracasei CBA L74 Metabolic Products and Fermented Milk for Infant Formula Have Anti-Inflammatory Activity on Dendritic Cells In Vitro and Protective Effects against Colitis and an Enteric Pathogen In Vivo
Source: PLoS One. 2014 Feb 10;9(2):e87615. doi: 10.1371/journal.pone.0087615 (PMC3919712; doi:10.1371/journal.pone.0087615)
Supplement: Table S1 — List of forward and reverse primers used for Q-PCR of the reported genes. (PDF) [file pone.0087615.s003.pdf]

Gene	Forward primer (5'->3')	Reverse primer (5'->3')
**Rpl32 (Ralp32)**	AAGCGAAACTGGCGGAAAC	TAACCGATGTTGGGCATCAG
**Tbet**	TCAACCAGCACCAGACAGAG	CCACATCCACAAACATCCTG
**Gata3**	GATGTAAGTCGAGGCCCAAG	AGGCATTGCAAAGGTAGTGC
**Ifn-g**	CACGGCACAGTCATTGAAAG	GCTGATGGCCTGATTGTCTT
**Il-17a**	AAGAAGCAGCCATTGGAGAA	TGCTACCTCCCTCAGAATGG
**Il-6**	CCATAGCTACCTGGAGTACATG	TGGAAATTGGGGTAGGAAGGAC
**Il33**	CCCGCCTTGCAAAATAAGA	CTTATGGTGAGGCCAGAACG
**Il-1b**	GACCTTCCAGGATGAGGACA	TCCATTGAGGTGGAGAGCTT
**Cxcl1 (Kc)**	GCTGGGATTCACCTCAAGAA	TCTCCGTTACTTGGGGACAC
**Ccl2**	CACGTGTTGGCTCAGCCAGATGC	CCTTCTTGGGGTCAGCACAGACC
**Cox2**	CCACTTCAAGGGAGTCTGGA	AGTCATCTGCTACGGGAGGA
**Nos2 (iNos)**	TCTCAAACTGCTCTGAGGTG	CGTTGGATTTGGAGCAGAAGTG
**Ahr**	TAGGCTCAGCGTCAGCTACC	TGCCAGTCTCTGATTTGTGC
**Indo**	AGAGCTCGCAGTAGGGAACAG	CATCACCATGGCGTATGTG
**Tjp1 (Zo-1)**	CGCGGAGAGAGACAAGATGT	CCTGTGAAGCGTCACTGTGT

Supplementary Table 1 Primers list
